# The pharmacogGENEtics in Youth Depression (GENE-YD) study for personalizing antidepressant pharmacotherapy for young Western Australians – A feasibility study

**DOI:** 10.1038/s41398-026-04321-x

**Published:** 2026-08-01

**Authors:** Bradley Roberts, Zahra Cooper, Aleksandra Miljevic, Susanne Stanley, Bernadette T. Majda, Khan R. L. Collins, Sarah Woodland, Michael J. Kyron, Svetlana Baltic, Suzanna E. L. Temple, P. Anthony Akkari, Sean D. Hood, Jennifer Rodger

**Affiliations:** 1 Perron Institute for Neurological and Translational Science, Nedlands, WA Australia; 2 School of Biological Sciences, The University of Western Australia, Crawley, WA Australia; 3 School of Biomedical Sciences, The University of Western Australia, Crawley, WA Australia; 4 Division of Psychiatry, School of Medicine, The University of Western Australia, Crawley, Australia; 5 Medical School, Curtin University, Bentley, WA Australia; 6 North Metropolitan Health Service, Western Australian Department of Health, Nedlands, WA Australia; 7 Rural Clinical School, School of Medicine, The University of Western Australia, Crawley, Australia; 8 Suicide Prevention and Resilience Research Centre, School of Psychological Science, The University of Western Australia, Crawley, WA Australia; 9 Gene S Pty Ltd, Perth, WA Australia; 10 Insitute for Respiratory Health, Nedlands, WA Australia; 11 School of Clinical Medicine, University of New South Wales, Kensington, NSW Australia; 12 School of Human Sciences, The University of Western Australia, Crawley, WA Australia; 13 Personalised Medicine Centre, Murdoch University, Murdoch, WA Australia; 14 Division of Neurology, Duke University Medical Centre, Duke University, Durham, NC USA

**Keywords:** Predictive markers, Clinical genetics

## Abstract

Pharmacogenetic (PGx) testing to guide antidepressant prescribing is becoming increasingly available. However, its uptake into routine psychiatric practice for young people remains limited due to insufficient youth-specific evidence and uncertainty regarding optimal trial design and outcome selection. This pilot study aimed to evaluate the feasibility of implementing a rigorous triple-blind randomized controlled trial to examine PGx-guided antidepressant treatment compared with treatment-as-usual (TAU) in young Australians with depression. The pharmacoGENEtics in Youth Depression (GENE-YD) study recruited young people aged 16–24 years old with major depressive disorder over 13 months, allocating 1:1 to PGx-Guided treatment or TAU. Depressive symptoms and quality-of-life outcomes were measured using rater-administered measures and self-report questionnaires at Baseline and at 6- and 12-weeks follow-up. Feasibility outcomes included recruitment, adherence, attrition, participant satisfaction, and protocol fidelity. Of 73 individuals screened, 55 were eligible and randomized. Baseline data were collected from 39 participants, with 38 completing assessments through Week 12. Adherence to the study protocol and assessment schedule was high, attrition was low, and participant satisfaction was strong. Clinically meaningful improvements were observed, with the PGx-Guided group showing greater reductions in depressive symptom severity, higher rates of treatment response and remission, and improved quality-of-life outcomes compared with TAU. The GENE-YD Study demonstrated that a triple-blind PGx-guided antidepressant trial is feasible and acceptable in young people with depression, with preliminary indications of clinical benefit. These findings support the inclusion of broader patient-centered outcomes beyond symptom change and will inform methodological refinements for future fully powered youth-specific PGx trials.

## Introduction

Early and effective antidepressant treatment is vital during adolescence and young adulthood, as these developmental periods are critical for cognitive and social maturation [1]. Untreated or inadequately treated depression during this period can have long-term impacts on overall health and quality of life [2]. In the past decade, youth aged 24 years and under have shown the highest rate of increase in antidepressant prescription and usage [3]. However, evidence from controlled trials shows almost 40% of youth fail to respond to their initial antidepressant pharmacotherapy [4, 5]. As a result, young people often undergo multiple dose adjustments and medication switches, prolonging distress and increasing the risk of functional impairment, disengagement from treatment, and suicidality [6–9]. Such clinical evidence has led to growing interest in personalized medicine for young people [10], with pharmacogenetics emerging as a promising approach [11–13].

Pharmacogenetic (PGx) testing examines how inherited genetic variation influences individual differences in drug metabolism, efficacy, and risk of adverse effects, enabling tailored antidepressant selection and dosing [14]. In the broader PGx literature, PGx-guided prescribing has shown modest heterogeneous outcomes [15], likely reflecting differences in study design, clinical populations, antidepressant selection, and PGx panels. To date, randomized controlled trials (RCTs) evaluating PGx-guided antidepressant prescribing specifically in young people remain limited. A large trial in adolescents (13–18-year-olds) in the United States saw no difference in treatment outcomes between PGx-guided and treatment-as-usual groups [16]. However, post-hoc analyses stratifying participants by gene-drug interaction risk demonstrated a reduced burden of medication-related side effects for individuals with a low/medium interaction when compared to high [17]. Conversely, a recent RCT in China reported marked improvement in PGx-guided group outcomes compared to control, but only in adolescents (14–18-year-olds) and young adults (18–35-year-olds) [18], highlighting the importance of age-specific trials in generating robust evidence to guide implementation.

These prior RCTs emphasize the need to expand the limited evidence base for PGx-guided antidepressant prescribing in youth and offer insights to improve future trials, including incorporating outcomes beyond symptom change such as quality of life [19], and the implementation of a rigorous triple-blinded RCT design to minimize prescribing clinician biases [16]. Given the considerable cost, time, and complexity associated with a fully powered triple-blind RCT, a feasibility study designed to test the protocol and its implementation represents an appropriate and necessary next step.

This pilot study aimed to investigate the feasibility of the pharmacoGENEtics in Youth Depression (GENE-YD) study protocol, a triple-blind RCT, evaluating PGx-guided intervention for antidepressant prescription in youth. The specific objectives were to (i) evaluate recruitment feasibility, (ii) assess participant adherence and attrition rates, (iii) evaluate participant satisfaction with recruitment, intervention delivery, and data collection procedures, (iv) explore preliminary between-group effects in outcomes measures over time, and (v) identify variables to refine power calculations for a larger RCT. Although conducted within an Australian context, the challenges addressed in this study, including trial-and-error antidepressant prescribing, early intervention in youth, and the need for more individualized treatment approaches, are widely recognized, supporting the broader relevance of this work and the applicability of this novel trial design in international settings.

## Method

### Study design and oversight

This study was a triple-blind RCT conducted at the Perron Institute for Neurological and Translational Science, Perth, Western Australia between July 2024 and December 2025, approved by the Department of Health, Western Australia’s Human Research Ethics Committee (RGS0000006822) and recognized by the University of Western Australia’s Human Research Ethics Committee (2024/ET000685). Full details of the protocol and procedures are reported in the published study protocol [20], and all study protocols and amendments are available at the Australian New Zealand Clinical Trials Registry (ACTRN12624000760572). All participants provided written informed consent prior to participation.

### Participants

Eligible participants were adolescents and young adults aged 16–24 years with a diagnosis of major depressive disorder (MDD), either first-episode or relapsed, as defined by the Mini International Neuropsychiatric Interview 7.0.2 for Diagnostic and Statistical Manual of Mental Disorders, Fifth Edition (DSM-5 M.I.N.I.). Exclusion criteria included significant suicidal risk based on the Montgomery-Åsberg Depression Rating Scale (MADRS) and/or DSM-5 M.I.N.I., signs of a substance use disorder not in remission (other than nicotine or caffeine), as determined during DSM-5 M.I.N.I. screening assessment, a diagnosis with a concurrent psychiatric diagnosis of any one or more of the following: bipolar disorder, psychotic disorders (i.e., psychotic MDD, schizophrenia, schizoaffective disorder, schizophreniform disorder), or cognitive disorders (i.e., intellectual impairment/dementia), as determined by participant medical history or during DSM-5 M.I.N.I. screening assessment, the failure of two or more prior adequate trials of evidenced-based pharmacotherapy treatments for MDD, reports of a history of significant hepatic or renal disease affecting drug metabolism, and pregnant or breast-feeding women at the time of enrolment.

### Interventions

Participants were randomized 1:1 to receive antidepressant pharmacotherapy tailored to their PGx profile (PGx-Guided group), or based upon current best practice recommendations (Control group: treatment-as-usual; TAU), informed by the Royal Australian and New Zealand College of Psychiatrists clinical practice guidelines for mood disorders [21]. Randomization was conducted using a computerized adaptive randomization procedure (minimization method) by an independent member of the research team. PGx testing utilized the next generation sequencing ‘RosaMind’ PGx Solution developed by, *Gene S* (Perth, Western Australia) [22], assessing key pharmacogenes involved in antidepressant metabolism and response (i.e., *CYP2D6*, *CYP2C19*; Supplementary Material S1). All participants and their treatment team, along with study assessors, were blinded to group allocation throughout the duration of the trial period. Further information on the study protocol and procedures can be found in the published study protocol [20].

Treatment Guides written by an unblinded psychiatrist on the research team and informed by participants’ demographic, clinical, and PGx information, were provided to participants’ prescribing clinicians who were encouraged to follow the recommendations provided on the Treatment Guide (Supplementary Material S2), whilst using their clinical judgement on what was appropriate for their patient. Participants were required to initiate or modify antidepressant treatment to complete Baseline assessment and be included in the analytic sample. Treatment decisions were encouraged to be made collaboratively between the participant and their prescribing clinician, and participants who did not wish to proceed with medication initiation or change did not continue beyond randomization.

Participants were assessed at drug prescription (Baseline) and again at 6- and 12-weeks following treatment intervention. Fortnightly informal calls at Weeks 2, 4, 8, and 10 were conducted to check-in on participants and their mental health and wellbeing. Feedback interviews were scheduled 24–28 weeks from Baseline.

### Outcomes

Primary feasibility outcomes were to estimate the proportion of voluntary participants that were eligible and who consented to participate in a larger-scaled RCT. Further primary outcomes were to estimate adherence and attrition to trial protocols and study procedures and assess the acceptability and participant satisfaction of the GENE-YD protocol, methodology of recruitment, intervention delivery, and data collection. Secondary outcomes included assessing trends in measures of depression symptomology (MADRS, QIDS-SR16) and quality-of-life (DASS-21, AQoL-8D, ASEC, MARS-5, LCQ, WPAI) evaluated at Baseline, Week 6, and Week 12, and identifying variables to refine power calculations for a larger sample. Definitions and measurement procedures are detailed in the published protocol [20].

### Target sample size

As this study was designed as a pilot RCT with feasibility as the primary outcome, a target sample size of a minimum of 20 participants per group completing the study was specified a priori [20]. To achieve this, approximately 80 participants were planned to be recruited to account for anticipated attrition and ineligibility, including participants not meeting DSM-5 M.I.N.I. criteria or not proceeding to treatment initiation. This sample size was consistent with recommendations for pilot RCTs and aligned with previous PGx pilot studies [23, 24].

### Statistical analyses

Analyses followed the prespecified statistical plan described in the published protocol [20]. Primary feasibility outcomes (recruitment, adherence, and attrition) were summarized using descriptive statistics and recruitment proportions. As a pilot feasibility study, analyses of secondary clinical outcomes were exploratory and not powered to detect definitive between-group differences. In line with recommendations for pilot trial analyses [25–27], findings are reported as estimate effect sizes and confidence intervals and presented to characterize trends and inform the design of a future definitive trial. All analyses were conducted on a modified intention-to-treat basis, including all participants who initiated or modified antidepressant treatment and completed Baseline assessment. Participants with missing data due to withdrawal or loss to follow-up before the 6-week post-intervention assessment, were classified as non-responders and non-remitters. Mixed-effects models assessed changes in depression severity, quality-of-life, and related measures over time, with treatment groups as the between-participant factor and a random intercept for participant to account for repeated measures. Continuous outcomes were modelled using linear mixed-effects models with an identity link. All available data is under a missing at random assumption. Descriptive statistics and visualizations were used to illustrate symptom trajectories. Baseline characteristics of the included participants were described as frequencies and means with standard deviation. Outcomes are presented as means with standard error and observed mean change from Baseline to Week 6 and Week 12 follow-ups with 95% confidence intervals. Descriptive Baseline characteristics were broadly comparable between groups, and thus, primary models included treatment group as the sole predictor. As an exploratory sensitivity analysis, Bayesian mixed-effects models adjusting for baseline values of the outcome measures were also fitted to account for any baseline group differences (Supplementary Material S3). Independent analyses of secondary outcomes were undertaken by MJK, who was independent from the research team and blinded to treatment allocation. Independent analyses were conducted without prior knowledge of the analytical decisions made by the primary research team, to minimize potential bias.

## Results

### Eligibility and recruitment of participants

Due to insufficient recruitment, the study was terminated prematurely, and the targeted minimum sample size of 40 participants was not reached. A total of 184 potential participants received a pre-screen call, of whom 73 were followed up with an in-person screening appointment to assess eligibility by a study investigator. Of these, 55 participants passed eligibility and were enrolled into the GENE-YD protocol, receiving a PGx test, group allocation, and a Treatment Guide provided to their nominated general practitioner (GP). Sixteen of the eligible participants did not go ahead with a medication change as per the Treatment Guide (29.1%), and a final sample of 39 participants received an antidepressant medication from their Treatment Guide and completed the Baseline assessments.

**Fig. 1:**
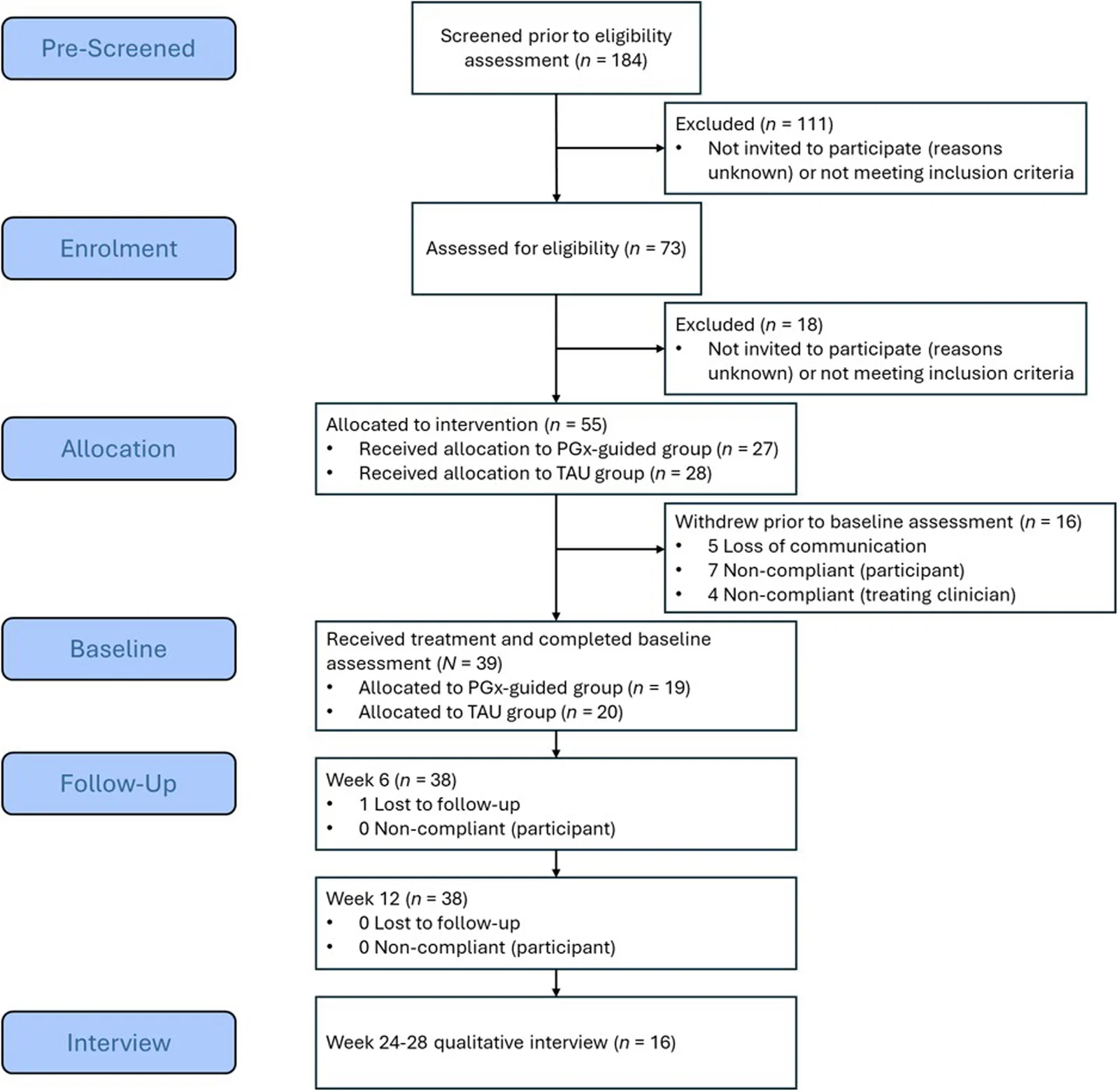
CONSORT flow diagram of participants through the pharmacoGENEtics in Youth Depression (GENE-YD) study. This flow diagram includes screening, eligibility assessment, allocation, Baseline completion, follow-up timepoints, and qualitative interview participation.

Of the Baseline assessed sample (*N* = 39), 38 completed follow-up assessments at Week 6 (97.4%) and, again at Week 12 (97.4%). Of those who completed the Week 12 assessments, 16 completed a feedback interview between Weeks 24–28 (42.1%). The flow of participants and follow-up rates are shown in Fig. 1.

### Cohort characteristics

In the full sample, the mean age was 20.5 years (SD 2.1, range 17–24), 24 participants were assigned female at birth (61.5%), 16 were born outside Australia (41.0%), and 25 identified as White/Caucasian descent (64.1%). Seventeen participants were previously diagnosed with depression (43.6%), 16 with generalized anxiety disorder (41.0%), and 28 had undergone or were currently undergoing antidepressant pharmacotherapy prior to participating in this study (71.8%). Of those 28, 12 had previously failed one antidepressant pharmacotherapy treatment or were looking to change their current medication due to either a lack of efficacy or the presentation of side effects (42.9%). Over half reported a family history of a psychiatric disorder (51.3%), with 13 participants reporting a family history of depression (33.3%). Baseline demographic characteristics were broadly similar across treatment groups (Table 1).

**Table 1:** Baseline characteristics for each interventional group, PGx-Guided and Treatment-as-Usual, and the whole cohort.

	Total	Intervention Groups	
	All (*N* = 39)	TAU (*n* = 20)	PGx-Guided (*n* = 19)
*n* (%)	39 (100)	20 (51.3)	19 (48.7)
Age (yrs), mean (SD)	20.5 (2.1)	20.4 (2.0)	20.7 (2.2)
Female sex at birth, *n* (%)	24 (61.5)	12 (60.0)	12 (63.2)
Ethnicity, *n* (%)			
Caucasian	25 (64.1)	14 (70.0)	11 (57.9)
Asian	12 (30.8)	5 (25.0)	7 (36.8)
Mixed Race	2 (5.1)	1 (5.0)	1 (5.3)
Australian born, *n* (%)	23 (59.0)	12 (60.0)	11 (57.9)
Student status, *n* (%)			
University / Other higher education	32 (82.1)	15 (75.0)	17 (89.8)
TAFE / Technical / Vocational / Registered training organization	1 (2.6)	0 (0.0)	1 (5.3)
Currently not studying	6 (15.4)	5 (25.0)	1 (5.3)
Employment status, *n* (%)			
Part-time employment	21 (53.8)	7 (35.0)	14 (73.7)
Self-employed – not employing others	2 (5.1)	2 (10.0)	0 (0)
Unemployed – seeking full-time employment	1 (2.6)	1 (5.0)	0 (0)
Unemployed – seeking part-time employment	7 (17.9)	6 (30.0)	1 (5.3)
Unemployed – not seeking employment	8 (20.5)	4 (20.0)	4 (21.1)
Living Situation, *n* (%)			
Family house or unit	27 (69.2)	12 (55.0)	15 (78.9)
Privately rented house or unit	6 (15.4)	4 (20.0)	2 (10.5)
Group home	1 (2.6)	1 (5.0)	0 (0.0)
Boarding house	5 (12.8)	3 (15.0)	2 (10.5)
Housing Satisfaction, *n* (%)			
Poor	1 (2.6)	1 (5.0)	0 (0.0)
Fair	7 (17.9)	3 (15.0)	4 (21.1)
Good	13 (33.3)	6 (30.0)	7 (36.8)
Very good	11 (28.2)	7 (35.0)	4 (21.1)
Excellent	7 (17.9)	3 (15.0)	4 (21.1)
Clinical depression (yes), *n* (%)	17 (43.6)	9 (45.0)	8 (42.1)
Clinical anxiety (yes), *n* (%)	16 (41.0)	8 (40.0)	8 (42.1)
Number of comorbidities, *n* (%)			
None	9 (23.1)	6 (30.0)	3 (15.8)
1–2	20 (51.3)	10 (50.0)	10 (52.6)
3–5	10 (25.7)	4 (20.0)	6 (31.6)
Current use of antidepressant medication (yes), *n* (%)	25 (64.1)	11 (55.0)	14 (73.7)
Previous use of antidepressant medication (yes), *n* (%)	28 (71.8)	14 (70.0)	14 (73.7)
Previous antidepressant medication failure (yes), *n* (%)	12 (30.8)	6 (30.0)	6 (31.6)
Family history of a psychiatric disorder (yes), *n* (%)	20 (51.3)	10 (50.0)	10 (52.6)
Family history of clinical depression (yes), *n* (%)	13 (33.3)	6 (30.0)	7 (36.8)
Perceived physical health, n (%)			
Poor	4 (10.3)	2 (10.0)	2 (10.5)
Fair	14 (35.9)	7 (35.0)	7 (36.8)
Good	13 (33.3)	7 (35.0)	6 (31.6)
Very good	7 (17.9)	3 (15.0)	4 (21.1)
Excellent	1 (2.6)	1 (5.0)	0 (0.0)
Study prescription, *n* (%)			
Citalopram	2 (5.1)	2 (10.0)	0 (0.0)
Escitalopram	8 (20.5)	5 (25.0)	3 (15.8)
Fluoxetine	7 (17.9)	2 (10.0)	5 (26.3)
Fluvoxamine	8 (20.5)	2 (10.0)	6 (31.6)
Sertraline	11 (28.2)	8 (40.0)	3 (15.8)
Mirtazapine	3 (7.7)	1 (5.0)	2 (10.5)

### Adherence with study protocol

Of the 39 participants who completed Baseline assessments (see Supplementary Material S4 for detailed Baseline assessment scores), 38 completed both rater-assessed and self-report assessments at Baseline, Week 6 and Week 12 (97.4%). One participant completed the Week 6 researcher-rated assessment but did not return the self-report assessments (2.6%). In general, adherence with the study protocol was consistent with minor attrition from Baseline to Week 12 (Table 2) and several wellbeing check-ins at Week 4 (4/39, 10.3%) and Week 8 (1/39, 2.6%) missed.

**Table 2:**
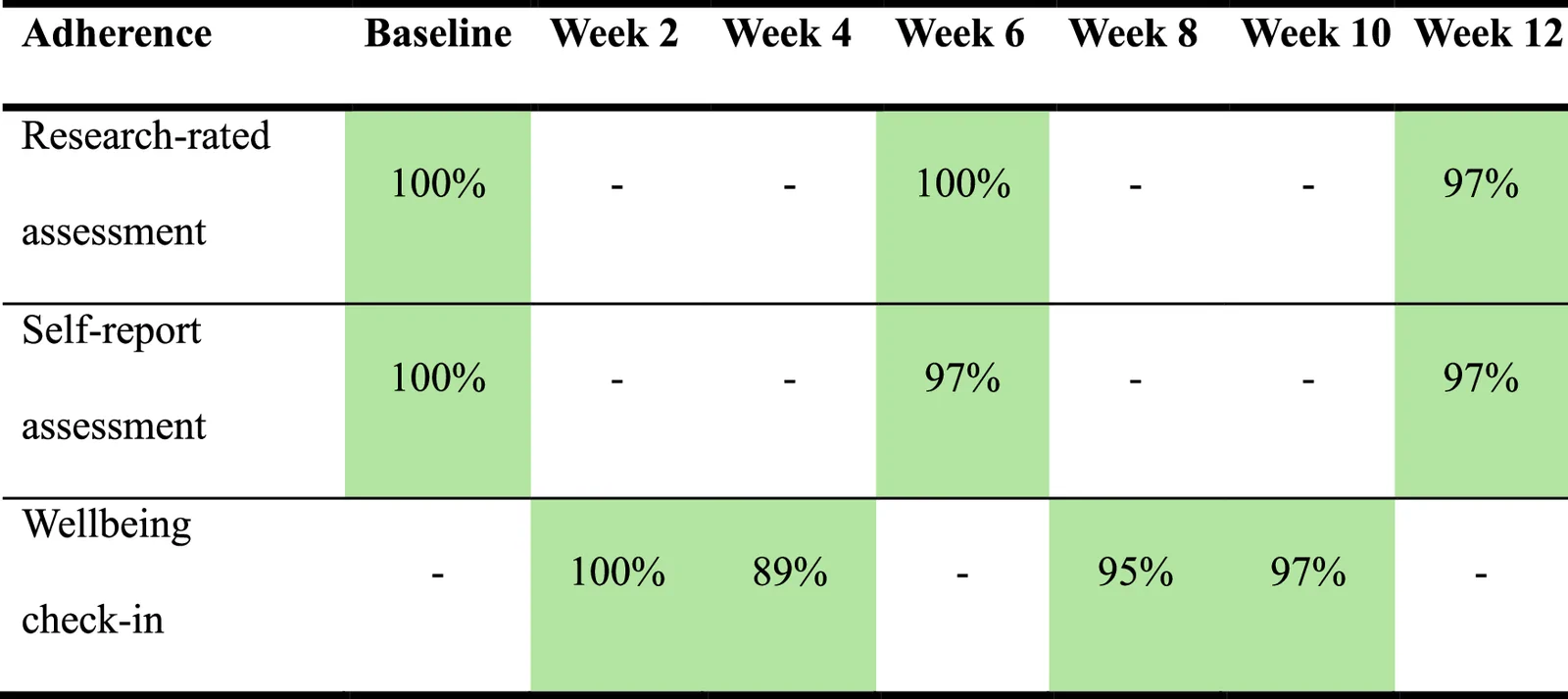
Proportion of participants (*N* = 39) who completed study assessments (Baseline, Week 6, Week 12) and wellbeing check-ins (Weeks 2, 4, 8, 10).

Note: Colour code: 80–100% green, 60–79% yellow, 0–59% red.

### Participant satisfaction with study protocol methodology

All participants who completed Week 12 were invited via text message to participate in a feedback interview in Weeks 24–28 to obtain participant satisfaction on the GENE-YD pilot protocol. Sixteen participants (42.1%) took part in these interviews, of which nine were from the PGx-Guided group and seven were from the TAU group.

All 16 participants reported a high level of satisfaction with the study methodology and their participation in the GENE-YD study. Most participants were recruited through study flyers (11/16, 68.8%) and word-of-mouth (3/16, 18.8%), and all described the enrolment-to-screening pathway, including study information dissemination and pre-screening procedures, as straightforward, well-explained, and easy to navigate. The study time commitment was widely viewed as manageable, and scheduled well-being check-in calls at Weeks 2, 4, 8, and 10 were generally perceived as helpful and contributed to a sense of support. A few participants noted that these wellbeing check-in calls could be enhanced with data collection using questionnaires with simple Likert-style questions. The primary friction point was the repetitive nature of the questions in the online self-report surveys, suggesting opportunities to reduce redundancy and streamline assessments.

*“It’s been a really good experience for me. I’m in a drastically better place now. Feels like I have a different brain in my head, you know, today versus 6 months ago.”* [GYD037]

Participants expressed no major concerns about the acceptability of PGx testing itself, but several participants highlighted that the usefulness of their results was constrained by minimal discussion or engagement with their GP, suggesting a need for stronger integration with primary care in future trials.

### Prescription congruency and treatment guide concordance

To characterize prescribing patterns and the influence of PGx information on clinical decision-making, we descriptively examined the congruency between prescribed antidepressants and participants’ PGx phenotypes at key timepoints. Congruency was not an outcome measure, and analyses were exploratory (see Supplementary Material S5 for definitions and analytic details).

At enrolment, 25 participants were taking an antidepressant medication. Prior to Baseline treatment changes, 57.1% (8/14) of participants in the PGx-Guided group and 54.5% (6/11) in the TAU group were prescribed medications congruent with their PGx phenotype. Following Baseline prescribing, the proportion of participants receiving congruent medication increased to 78.9% (15/19) in the PGx-Guided group, whilst remaining largely unchanged in the TAU group (11/20, 55.0%). By Week 12, medication congruency decreased slightly in the PGx-Guided group (14/19, 73.7%) and increased slightly in the TAU group (12/20, 60.0%).

Prescription patterns were otherwise similar across groups, with 92.3% (36/39) of participants receiving an SSRI (Table 2). Prescribing concordance with the respective Treatment Guides was high, with 94.7% of PGx-Guided and 100% of TAU participants prescribed medications included in their guides. This high level of concordance indicates that the integrity of the intervention was maintained.

### Symptom improvement and rates of response and remission

**Fig. 2:**
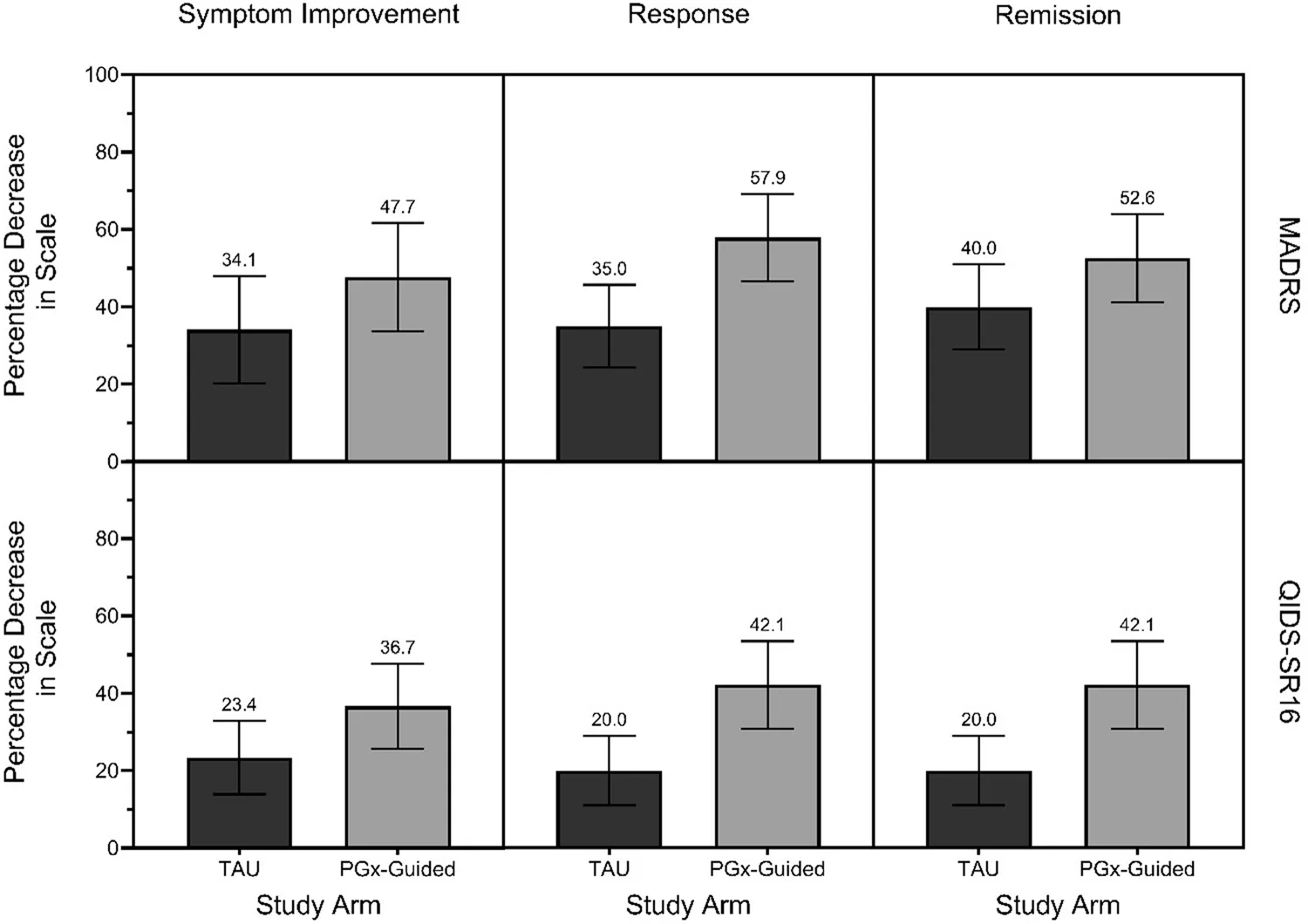
Symptom improvement, response, and remission rates at Week 12 among participants in the GENE-YD Study. Bars show the mean percentage of participants in the treatment-as-usual (TAU) and PGx-Guided groups for improvement, and who met criteria treatment response, or remission based on the MADRS and QIDS-SR16 depression assessments. Treatment response was defined as ≥50% reduction in scale score from Baseline, and remission was defined as Week 12 MADRS scores ≤10 and QIDS-SR16 scores ≤5. Error bars represent the standard error of the mean (SEM).

At Baseline, the mean MADRS scores were 25.8 (2.2) and 22.6 (2.2) for the PGx-Guided and TAU groups, respectively. By Week 12, both the PGx-guided and TAU groups showed reductions in depressive symptoms, with scores decreasing to 13.5 (2.2) and 14.8 (2.2), respectively. The magnitude of symptom change from Baseline to Week 12 was −12.3 (47.7, 95% CI [−18.5, −6.1]) for the PGx-Guided group and −7.7 (34.1, 95% CI [−13.9, −1.6]) for the TAU group (Fig. 2). The between-group difference in change from Baseline to Week 12 favored the PGx-guided group (Δ_PGx-TAU_ = −4.6, 95% CI [−13.4, 4.1]).

Baseline QIDS-SR16 scores averaged 12.8 (1.0) in both the PGx-Guided and TAU groups. At Week 6, mean scores had decreased to 9.6 (1.0) in the PGx-Guided group and 10.1 (1.0) in the TAU group. By Week 12, further reductions were observed in both the PGx-Guided group (8.1 [1.0]) and the TAU group (9.8 [1.0]), corresponding to change scores of −4.7 (36.7, 95% CI [−7.5, −2.0]) and −3.0 (23.4, 95% CI [−5.7, −0.3]), respectively (Fig. 2). Between-group differences in change from Baseline to Week 12, were small (Δ_PGx-TAU_ = −1.7, 95% CI [−5.6, 2.1]).

Rates of clinical response and remission were consistent with the symptom change data (Fig. 2). For the MADRS, the proportion of participants meeting criteria for treatment response (defined as ≥50% reduction in scale score from Baseline) was 57.9 and 35.0% in the PGx-Guided and TAU groups, respectively. Similarly, the proportion meeting remission criteria (defined as Week 12 MADRS scores ≤10) was 52.6% of PGx-Guided and 40.0% of TAU participants, respectively. For the QIDS-SR16, the proportion meeting both treatment response (defined as ≥50% reduction in scale score from Baseline) and remission (defined as Week 12 QIDS-SR16 scores ≤5) criteria was 42.1% of PGx-Guided and 20.0% of TAU participants, respectively.

Baseline-adjusted Bayesian analyses yielded effect estimates consistent with the primary analyses, with no substantive changes to the direction or interpretation of findings. Results were consistent across Bayesian models using alternative prior specifications (Supplementary Material S3).

### Outcome measures of quality of life

Table 3 provides a comprehensive overview of treatment group changes to quality-of-life measures over time. Overall, exploratory group differences were observed, with trends suggesting greater improvement in the PGx-Guided group.

**Table 3:** Self-reported quality of life measures over time for each scale and domain used within the GENE-YD Study.

			Timepoint					
Scale	Domain		Baseline	Week 6	Δ6	Week 12	Δ12	Between Group Δ12_PGx-TAU_
DASS-21	Depression	TAU (*n* = 20)	7.9 (0.9)	6.7 (0.9)	−1.2 (−3.7, 1.3)	6.6 (0.9)	−1.3 (−3.8, 1.2)	-
		PGx-Guided (*n* = 19)	8.3 (0.9)	7.4 (0.9)	−0.9 (−3.4, 1.6)	6.3 (0.9)	−2.0 (−4.5, 0.6)	−0.7 (−4.2, 2.9)
	Anxiety	TAU (*n* = 20)	7.3 (0.8)	5.5 (0.8)	−1.8 (−3.0, 0.4)	4.8 (0.8)	−2.5 (−4.6, −0.3)	-
		PGx-Guided (*n* = 19)	6.4 (0.8)	5.7 (0.8)	−0.7 (−3.0, 1.5)	3.8 (0.8)	−2.7 (−4.9, −0.5)	−0.2 (−3.3, 2.9)
	Stress	TAU (*n* = 20)	9.3 (0.8)	8.2 (0.9)	−1.1 (−3.5, 1.2)	7.3 (0.9)	−2.0 (−4.4, 0.3)	-
		PGx-Guided (*n* = 19)	9.3 (0.9)	8.2 (0.9)	−1.1 (−3.5, 1.2)	7.0 (0.9)	−2.3, −4.7, 0.0)	−0.3 (−3.7, 3.0)
AQoL-8D	Independent Living	TAU (*n* = 20)	8.5 (0.6)	8.2 (0.6)	−0.3 (−2.0, 1.4)	7.8 (0.6)	−0.7 (−2.3, 1.0)	-
		PGx-Guided (*n* = 19)	8.2 (0.6)	7.6 (0.6)	−0.5 (−2.2, 1.2)	7.5 (0.6)	−0.7 (−2.4, 1.0)	0.0 (−2.4, 2.4)
	Senses	TAU (*n* = 20)	5.5 (0.4)	4.8 (0.4)	−0.6 (−1.7, 0.5)	5.7 (0.4)	0.2 (−0.9, 1.3)	-
		PGx-Guided (*n* = 19)	5.8 (0.4)	5.8 (0.4)	0.0 (−1.2, 1.1)	5.8 (0.4)	0.0 (−1.3, 1.1)	−0.2 (−1.8, 1.3)
	Pain	TAU (*n* = 20)	6.2 (0.5)	5.5 (0.5)	−0.7 (−2.1, 0.8)	6.1 (0.5)	−0.1 (−1.5, 1.3)	-
		PGx-Guided (*n* = 19)	5.6 (0.5)	4.8 (0.5)	−0.8 (−2.3, 0.6)	4.9 (0.5)	−0.7 (−2.1, 0.8)	−0.6 (−2.6, 1.5)
	Mental Health	TAU (*n* = 20)	23.7 (0.9)	21.8 (0.9)	−1.9 (−4.4, 0.6)	20.5 (0.9)	−3.2 (−5.7, −0.5)	-
		PGx-Guided (*n* = 19)	23.6 (0.9)	21.5 (0.9)	−2.1 (−4.8, 0.4)	20.5 (0.9)	−3.1 (−5.8, −0.6)	0.1 (−3.6, 3.7)
	Happiness	TAU (*n* = 20)	12.0 (0.5)	11.5 (0.5)	−0.5 (−2.0, 1.0)	11.4 (0.5)	−0.6 (−2.1, 0.9)	-
		PGx-Guided (*n* = 19)	12.6 (0.5)	11.6 (0.5)	−1.0 (−2.5, 0.5)	11.0 (0.5)	−1.6 (−3.1, −0.1)	−1.0 (−3.1, 1.1)
	Self-Worth	TAU (*n* = 20)	8.8 (0.4)	7.9 (0.5)	−0.9 (−2.2, 0.3)	8.1 (0.5)	−0.7 (−2.0, 0.5)	-
		PGx-Guided (*n* = 19)	9.3 (0.5)	8.4 (0.5)	−0.9 (−2.2, 0.4)	7.8 (0.5)	−1.5 (−2.8, −0.3)	−0.8 (−2.6, 1.0)
	Coping	TAU (*n* = 20)	9.0 (0.4)	8.4 (0.4)	−0.6 (−1.8, 0.7)	8.2 (0.4)	−0.8 (−2.0, 0.5)	-
		PGx-Guided (*n* = 19)	9.5 (0.4)	9.2 (0.4)	−0.3 (−1.5, 0.9)	8.2 (0.4)	−1.3 (−2.5, −0.1)	−0.5 (−2.3, 1.1)
	Relationships	TAU (*n* = 20)	17.2 (0.9)	16.2 (1.0)	−1.0 (−3.6, 1.6)	15.7 (1.0)	−1.5 (−4.1, 1.1)	-
		PGx-Guided (*n* = 19)	17.2 (1.0)	15.4 (1.0)	−1.8 (−4.5, 0.8)	14.8 (1.0)	−2.4 (−5.1, 0.2)	−0.9 (−4.6, 2.8)
	Total AQoL-8D Score	TAU (*n* = 20)	90.7 (3.5)	84.3 (3.6)	−6.4 (−16.2, 3.4)	83.4 (3.6)	−7.3 (−17.1, 2.5)	-
		PGx-Guided (*n* = 19)	91.9 (3.6)	84.3 (3.6)	−7.6 (−17.6, 2.3)	80.5 (3.6)	−11.4 (−21.3, −1.5)	−4.1 (−18.1, 9.8)
ASEC	Frequency	TAU (*n* = 20)	3.9 (0.8)	5.4 (0.8)	1.5 (−0.7, 3.8)	6.2 (0.8)	2.4 (0.1, 4.6)	-
		PGx-Guided (*n* = 19)	2.4 (0.8)	6.5 (0.8)	4.1 (1.8, 6.4)	4.6 (0.8)	2.2 (−0.1, 4.4)	−0.2 (−3.4, 3.0)
	Severity	TAU (*n* = 20)	5.5 (0.9)	4.2 (0.9)	−1.3 (−3.7, 1.2)	4.3 (0.9)	−1.2 (−3.6, 1.3)	-
		PGx-Guided (*n* = 19)	3.8 (0.9)	3.5 (0.9)	−0.3 (−2.8, 2.2)	2.5 (0.9)	−1.3 (−3.7, 1.2)	−0.1 (−3.6, 3.3)
MARS-5	Medication Adherence	TAU (*n* = 20)	22.6 (0.5)	23.1 (0.6)	0.5 (−1.0, 2.0)	22.6 (0.6)	0.0 (−1.5, 1.5)	-
		PGx-Guided (*n* = 19)	21.8 (0.8)	22.6 (0.6)	0.8 (−0.7, 2.4)	21.9 (0.6	0.1 (−1.4, 1.6)	0.1 (−2.0, 2.3)
LCQ	Social Participation	TAU (*n* = 20)	18.4 (2.9)	-	-	21.4 (3.0)	3.0 (−5.1, 11.2)	-
		PGx-Guided (*n* = 19)	9.7 (3.0)	-	-	15.8 (3.0)	6.1 (−2.2, 14.4)	3.0 (−8.6, 14.7)
	Study Participation	TAU (*n* = 20)	17.1 (3.4)	-	-	7.6 (3.5)	−9.8 (−18.9, 0.0)	-
		PGx-Guided (*n* = 19)	7.6 (3.5)	-	-	10.9 (3.5)	3.3 (−6.3, 12.8)	12.7 (−0.7, 26.2)
	Unpaid Work Participation	TAU (*n* = 20)	0.6 (0.6)	-	-	1.5 (0.6)	1.0 (−0.7, 2.6)	-
		PGx-Guided (*n* = 19)	0.6 (0.6)	-	-	0.4 (0.6)	−0.2 (−1.9, 1.4)	−1.2 (−3.5, 1.1)
	Carer Participation	TAU (*n* = 20)	0.7 (4.2)	-	-	4.0 (5.2)	3.3 (−9.9, 16.4)	-
		PGx-Guided (*n* = 19)	8.7 (4.3)	-	-	1.4 (6.6)	−7.3 (−22.8, 8.2)	−10.6 (−30.9, 9.8)
	Paid Work Participation	TAU (*n* = 20)	6.8 (2.4)	-	-	14.2 (3.2)	7.5 (−0.4, 15.4)	-
		PGx-Guided (*n* = 19)	15.2 (2.5)	-	-	22.0 (2.9)	6.8 (−0.6, 14.2)	−0.7 (−11.5, 10.1)
	GP Visits: Annually	TAU (*n* = 20)	7.4 (1.4)	-	-	7.5 (1.4)	0.1 (−3.6, 4.0)	-
		PGx-Guided (*n* = 19)	7.6 (1.4)	-	-	6.3 (1.4)	−1.3 (−5.2, 2.5)	−1.5 (−6.9, 3.9)
WPAI	Absenteeism	TAU (*n* = 20)	0.4 (2.8)	-	-	5.5 (2.6)	5.1 (−2.3, 12.6)	-
		PGx-Guided (*n* = 19)	5.0 (2.2)	-	-	3.0 (2.1)	−2.0 (−8.0, 4.0)	−7.2 (−16.7, 2.4)
	Presenteeism	TAU (*n* = 20)	38.9 (6.9)	-	-	35.0 (6.6)	−3.9 (−22.6, 14.8)	-
		PGx-Guided (*n* = 19)	30.7 (5.5)	-	-	18.0 (5.4)	−12.7 (−27.9, 2.4)	−8.8 (−32.9, 15.2)
	Work Impairment	TAU (*n* = 20)	29.1 (7.5)	-	-	33.6 (6.8)	−5.5 (−25.4, 14.4)	-
		PGx-Guided (*n* = 19)	33.7 (6.0)	-	-	20.3 (5.8)	−13.4 (−29.9, 3.0)	−8.1 (−33.7, 17.8)
	Activity Impairment	TAU (*n* = 20)	42.6 (5.8)	-	-	34.4 (6.0)	−8.2 (−24.5, 8.1)	-
		PGx-Guided (*n* = 19)	40.0 (6.0)	-	-	27.2 (6.0)	−12.8 (−29.3, 3.7)	−4,6 (−27.8, 18.6)

Timepoint measures presented as group means (SEM), change (Δ) to baseline, and between group as mean difference (95% CI).

#### Depression, anxiety, and stress

The DASS-21 showed variable changes over the 12-week protocol. Both groups exhibited a moderate decrease in mean depression from Baseline to Week 12 (Δ_PGx_ = −2.0, 95% CI [−4.5, 0.6]; Δ_TAU_ = −1.3, 95% CI [−3.8, 1.2]). Similarly, mean anxiety decreased over time, with a reduction by Week 12 in both the PGx-Guided group (Δ_PGx_ = −2.7, 95% CI [−4.9, −0.5]) and the TAU group (Δ_TAU_ = −2.5, 95% CI [−4.6, −0.3]). Mean stress scores showed moderate decreases in both groups (Δ_PGx_ = −2.3, 95% CI [−4.7, 0.0]; Δ_TAU_ = −2.0, 95% CI [−4.4, 0.3]). Small between group differences were observed for all measures (Depression: Δ_PGx-TAU_ = −0.7, 95% CI [−4.2, 2.9]; Anxiety: Δ_PGx-TAU_ = −0.2, 95% CI [−3.3, 2.9]; Stress: Δ_PGx-TAU_ = −0.3, 95% CI [−3.7, 3.0]).

#### Quality of life

Four domains of the AQoL-8D showed changes over time within and between groups. Lower scores on the AQoL-8D indicate better quality of life and therefore decreases over time reflect improvement. *Mental Health* decreased in both the PGx-Guided and TAU groups over time (Δ_PGx_ = −3.2, 95% CI [−5.8, −0.6]; Δ_TAU_ = −3.2, 95% CI [−5.7, −0.5]), whilst the PGx-guided group alone saw improvements to *Happiness* (Δ_PGx_ = −1.6, 95% CI [−3.1, −0.1]), *Self-Worth (*Δ_PGx_ = −1.5, 95% CI [−2.8, −0.3]), and *Coping* (Δ_PGx_ = −1.3, 95% CI [−2.5, −0.1]). Overall *Quality of Life* scores were decreased in the PGx-Guided group (Δ_PGx_ = −11.4, 95% CI [−21.3, −1.5]) compared to the TAU group (Δ_TAU_ = −7.3, 95% CI [−17.1, 2.5]), indicating an improved overall quality of life for individuals with PGx-Guided treatment.

#### Side effects

Whilst participants in the PGx-Guided group reported an increase in side-effect frequency from Baseline (2.4 [0.8]) to Week 6 (6.5 [0.8]) before a return towards Baseline at Week 12 (4.6 [0.8]), participants in the TAU group reported an increasing trend from Baseline (3.9 [0.8]) to Week 6 (5.4 [0.8]) and again to Week 12 (6.2 [0.8]). Side effect severity was lower in the PGx-Guided group across all timepoints when compared to the TAU group (Baseline PGx = 3.8 [0.9], TAU = 5.5 [0.9]; Week 6 PGx = 3.5 [0.9], TAU = 4.2 [0.9]; Week 12 PGx = 2.4 [0.9], TAU = 4.3 [0.9]).

#### Medication adherence

Medication adherence remained stable over the 12-week protocol in both groups, with negligible changes in both the PGx-Guided and TAU groups (Δ_PGx_ = 0.1, 95% CI [−1.4, 1.6]; Δ_TAU_ = 0.0, 95% CI [−1.5, 1.5]).

#### Community engagement

Community engagement, measured by the Living in the Community Questionnaire, showed variability over the 12-week protocol with minimal differences between groups*. Study Participation* remained relatively unchanged in the PGx-Guided group (Δ_PGx_ = 3.3, 95% CI [−6.3, 12.8]) whilst being slightly reduced from Baseline to Week 12 in the TAU group (Δ_TAU_ = −9.8, 95% CI [−18.9, 0.0]).

#### Work productivity impairment

Changes in the Work Productivity and Activity Impairment Questionnaire reports from Baseline to Week 12 showed an overall trend for a reduced impact of mental health on daily performance in the PGx-Guided group compared to the TAU group. The PGx-Group had a greater reduction in *Absenteeism* (Δ_PGx-TAU_ = −7.2, 95% CI [−16.7, 2.4]), *Presenteeism* (Δ_PGx-TAU_ = 8.8, 95% CI [−32.9, 15.2]), *Overall Work Productivity Loss* (Δ_PGx-TAU_ = −8.1, 95% CI [−33.7, 17.8]), and *Activity Impairment* (Δ_PGx-TAU_ = −4.6, 95% CI [−27.8, 18.6]).

### Gatekeeping analysis

A gatekeeping analysis was explored, in which participants with “normal” CYP2C19 and CYP2D6 PGx phenotypes were removed from analysis to examine only those with actionable variants for antidepressant response [28]. In this restricted sample (*N* = 36; *n*_*PGx*_ = 18, *n*_*TAU*_ = 18), the observed effect size for symptom change decreased from 0.33 to 0.31. Direction and magnitude of change over time in all outcome measures were generally consistent between the reduced sample and the full cohort (see Supplementary Material S6 for further details).

## Discussion

This pilot trial examined the feasibility and acceptability of implementing a triple-blind RCT protocol to evaluate PGx-guided antidepressant pharmacotherapy in young adults. Overall, findings indicated that the GENE-YD protocol was largely feasible, accepted by participants, and capable of detecting differential trends between study arms. Minor modifications are recommended for the design and optimization of a fully powered future RCT.

### Recruitment and enrolment of participants

Despite strong adherence and retention once enrolled, feasibility was limited by challenges in recruitment and early engagement. Participant flow into the GENE-YD study, averaged just over three participants per month across the 18-month recruitment period. Furthermore, nearly one-third of eligible individuals withdrew participation after enrolment and completion of PGx testing procedures but before commencing their study prescription and completing the Baseline assessments. This reduction from initial enrolment to Baseline assessment reflects challenges in transitioning participants to medication change, including participant-level factors such as evolving treatment preferences and initial misunderstandings regarding study requirements. This early attrition highlights the importance of refining participant information and consent procedures in future trials.

Challenges with recruiting young adults into psychopharmacology trials are well documented, with youth antidepressant trials commonly experiencing substantial early attrition during screening [4, 29, 30]. Our relatively low recruitment rate could potentially be due to reliance on flyer-based advertisement across tertiary education campuses and broader community outreach, limiting the study’s access to clinical populations actively seeking treatment. In addition, we experienced a relatively high early attrition rate, often due to either the participant or the participant’s prescribing GP being unwilling to change or initiate pharmacotherapy. Adding multiple methods of recruitment, including integration with clinical settings (inpatient and outpatient services) where adolescents and young adults are already seeking care, has been suggested to reduce early attrition and improve enrolment efficiency and may do so in a future fully powered RCT [16, 31]. Furthermore, raising GP awareness of study requirements before the prescribing appointment, may promote greater clinician engagement and adherence to the study protocol and promote discussion between participants and their GPs to improve primary care communication pathways and therapeutic relationships [32].

### Participant adherence to and satisfaction of the GENE-YD protocol

Adherence to the GENE-YD protocol was high, with participants completing 90% or more of the scheduled assessments over the 12-week period and treating GPs demonstrating high concordance with the assigned Treatment Guides. Given that adherence is a critical determinant of an intervention’s reach, efficacy, adoption, implementation, and maintenance [33], these findings demonstrate the practicality of the protocol in this population. Retention was similarly strong, with only one participant lost to follow-up, indicating that the overall study procedures were acceptable for young adults initiating antidepressant treatment.

The scheduled well-being check-in phone calls at Weeks 2, 4, 8, and 10 likely contributed to the high retention rate, as participants’ reports indicated that these brief contact points helped them remain engaged with the study procedures. While these contacts were not designed as a therapeutic intervention and were applied equally across groups, their supportive nature may have contributed to non-specific effects on outcomes. Participants’ reflections also suggested refinements to the protocol for a larger trial. Although overall satisfaction with the study structure was high, some participants identified the repetitive nature of content across some questionnaires as a source of mild burden. Minimizing redundancy and improving the efficiency of the survey battery was suggested to improve participatory satisfaction.

Finally, while the wellbeing check-ins were generally valued, several participants suggested that these contacts could be strengthened by including simple structured elements, such as simple Likert-style ratings, to complement open discussion around medication-related side effects. Such enhancements may allow future trials to capture more consistent interim data while preserving the supportive function of these calls.

### Data collection, patient characteristics, and outcomes

The study successfully captured repeated assessments of depressive symptom severity, quality of life, and side effect burden across the 12-week follow-up period. Data completeness was high, enabling reliable estimation of change trajectories despite the small sample. Baseline clinical characteristics were comparable to previous studies [16, 18], reflecting a group average moderate depression and a high rate of female participation. Characteristics were broadly similar between intervention groups, however some variation was observed, as expected in a pilot sample.

Across both arms, depressive symptoms and quality of life outcomes improved over time, consistent with expected effects of antidepressant initiation, but trends were generally more favorable in the PGx-Guided group. Specifically, PGx-guided pharmacotherapy was associated with greater reductions in depressive symptomology across both rater-assessed and self-report scales, alongside higher proportions meeting response and remission criteria. Similarly, quality of life domain-specific measures showed small to moderate directional differences favoring PGx, suggesting potential wider benefits beyond symptom reduction, impacting side effect burden, work performance, and self-esteem measures. However, the modest sample size means that these preliminary estimates should be interpreted with caution. A larger, adequately powered trial is needed to determine whether the favorable trends toward PGx-guided antidepressant pharmacotherapy observed here persist when sampling variability is reduced.

Beyond these broader considerations, the performance of specific outcome measures also provides guidance for refining the assessment battery in future work. The MARS-5, a brief self-report measure of medication adherence, did not detect meaningful differences between groups. This likely reflects uniformly high self-reported adherence across the sample rather than true behavioral equivalence, consistent with a Hawthorne effect whereby awareness of participation in a study and the possibility of receiving a potentially beneficial intervention enhances self-reported medication adherence [34]. Its limited sensitivity aligns with evidence from other clinical populations showing poor concordance between MARS-5 scores and objective adherence metrics, such as pharmacy refill data [35]. Similarly, the LCQ added little incremental value beyond existing measures of quality of life, functioning, and side-effect burden, largely mirroring information already captured by the AQoL-8D or the WPAI, suggesting it may be disused within a streamlined battery to improve participant experiences in the study. Finally, our findings suggest that the DASS-21 showed limited sensitivity to changes in depressive symptoms compared to the MADRS and QIDS-SR16, aligning with previous work indicating that the DASS-21 depression subscale may perform better as a broad screening tool than as an outcome measure in interventional studies [36, 37]. However, the DASS-21 successfully captured changes in anxiety, suggesting that if it were to be removed, an alternative self-report measure of anxiety should be considered to ensure adequate coverage of anxiety outcomes.

### Signal of efficacy

The pilot findings suggest a small-to-moderate directional benefit favoring PGx-Guided prescription, with an observed effect size of Cohen’s *d* = 0.33. Although the study was not powered for hypothesis testing, this effect size provides a useful estimate of the magnitude of change that might be expected in a larger trial. When used to model future trial design, this effect size translates to a required sample of approximately 292 participants to achieve 80% power with α = 0.05 in a definitive study. The current pilot had a post-hoc power of 0.17, which is consistent with its feasibility-focused design and indicates that the absence of confirmatory statistical signal should be interpreted as underpowered rather than a lack of effect.

In the gatekeeping analysis, participants with “normal” PGx phenotypes were removed to focus specifically on those with actionable variants for antidepressant response, consistent with the approach described by Swen et al. [28]. This restriction slightly reduced the sample size and, consequently, the statistical power of the analysis. While some trends appeared attenuated, this is likely an artifact of the reduced sample rather than a true change in the direction or magnitude of the effect. Taken together, these findings suggest a preliminary signal that may indicate potential benefit, but which requires confirmation in a fully powered trial.

### Future direction

Overall, the GENE-YD pilot study has led to several key modifications to the methodology for a subsequent RCT. Firstly, recruitment pathways should be supplemented with introducing study information within clinical settings where young adults typically begin antidepressant treatment. Secondly, the survey battery should be streamlined by reducing redundant measures. Thirdly, wellbeing check-ins should be enhanced by incorporating brief structured elements to complement open discussion, improve consistency of interim data, and maintain the supportive function valued by study participants. Considering the well-established comorbidity and similar pharmacotherapy treatment strategies between depression and anxiety [38–40], and the observed reduction in self-reported anxiety on the DASS-21 in this pilot, future recruitment strategies should broaden beyond a strict MDD diagnosis to also include young people with generalized anxiety disorder who are considering pharmacotherapy. Furthermore, future studies should incorporate objective measures of medication adherence, such as pharmacy refill data. These approaches may better reflect real-world clinical presentations, improve recruitment rates of a future trial, and enhance the applicability of PGx-guided antidepressant pharmacotherapy.

It is important to note that this pilot did not control for concurrent psychotherapy engagement or potential drug-drug-gene interaction effects arising from concomitant use of both psychotropic and non-psychotropic medications. Given the potential influence of these factors on treatment outcomes [41, 42], their absence represents a source of residual confounding. Future studies will benefit from accounting for these variables within the study design. Additionally, the generalizability of findings may be limited by the pilot nature and small sample size, and the recruitment strategy, which relied primarily on community advertising and tertiary education settings. Similarly, the exclusion of individuals with significant suicidal risk further limits generalizability, reflecting broader safety and ethical considerations in pilot and feasibility mental health trials [43]. As such, the sample may not fully represent the broader population of young people receiving mental health care, particularly those engaged in clinical services or with more complex and severe presentations [27]. Finally, Treatment Guides were prepared centrally by an unblinded psychiatrist without direct patient interaction. However, these recommendations were informed by detailed demographic and clinical information collected at screening, alongside participants’ PGx profiles. While prescribing clinicians retained discretion in final treatment decisions, this centralized approach may differ from typical clinical encounters involving an ongoing therapeutic relationship but was implemented as part of the study protocol to preserve real-world prescribing practices.

### Conclusion

The GENE-YD study protocol was feasible to deliver in adolescents and young adults commencing antidepressant pharmacotherapy, with high retention, good adherence, and generally positive participant experiences. Although exploratory, outcomes were more favorable for PGx-guided treatment across symptom, side effect, and quality-of-life measures compared to treatment-as-usual. This preliminary evaluation provides encouraging early evidence supporting the potential clinical value of personalized, PGx-guided antidepressant treatment for young people across Australia. Importantly, translation into routine care will depend not only on clinical effectiveness but also on broader implementation considerations, including cost, accessibility of testing, clinician engagement, and integration within existing healthcare systems. While findings must be interpreted in the context of a small, exploratory pilot, they support progression to a fully powered RCT to formally evaluate PGx-guided care within this broader implementation framework.

## Supplementary information

GENE-YD Supplementary Material

## Data Availability

All data generated or analyzed during this study are included in this published article and its supplementary material.
