# Supplementary material for: The pharmacogGENEtics in Youth Depression (GENE-YD) study for personalizing antidepressant pharmacotherapy for young Western Australians – A feasibility study
Source: Transl Psychiatry. 2026 Aug 1;16:574. doi: 10.1038/s41398-026-04321-x (PMC13680375; doi:10.1038/s41398-026-04321-x)
Supplement: Supplementary file 1 [file 41398_2026_4321_MOESM1_ESM.docx]

# *Supplementary Material*

This file contains the supplementary material for the manuscript titled “The PharmacoGENEtics in Youth Depression (GENE-YD) Study for personalizing antidepressant pharmacotherapy for young Western Australians – A feasibility study”. Contents are as follows:

- Supplementary Material S1. Gene S ‘RosaMind’ PGx Solution
- Supplementary Material S2. Example Treatment Guide
- Supplementary Material S3. Bayesian analyses
- Supplementary Material S4. Baseline assessment scores
- Supplementary Material S5. Prescription congruency
- Supplementary Material S6. Gatekeeper analysis

## Supplementary Material S1. *Gene S* ‘RosaMind’ PGx Solution

*Note: Genetic variations are analysed in the following pharmacogenes: ***CYP3A4***: partial gene coverage; ***CYP2D6***: whole gene; ***CYP2C9***: partial gene coverage (all exons); ***CYP2C19***: partial gene coverage (all exons); ***HLA-A***: whole gene; ***HLA-B***: whole gene.

**Note: HLA reporting only returns allele results and advice relevant to the indicated test (in mental health, HLA-A*31:01:01 and HLA-B*15:02:01).

The Gene S RosaMind PGx Test reports all CYP * alleles except:

- CYP2D6*27 which is reported as *1 (both are functionally the same).
- CYP2D6*74, *122, and *139 have unknown function and are reported as *1.
- CYP2D6*83 is non-functional and is reported as CYP2D6*36, also non-functional allele with the same exon 9 conversion and clinical impact.

This ensures our reports are accurate, consistent, and clinically meaningful.

The genetic variations detected by the *Gene S* RosaMind PGx Test have been validated for their association with drug metabolism, response, or adverse effects and published as per the guidelines of the Clinical Pharmacogenetics Implementation Consortium (CPIC) and the Royal Dutch Pharmacists Association, Pharmacogenetics Pharmacogenomics Working Group (DPWG). Only gene-drug associations with the highest level of evidence (Level 1 & 2A) and CPIC and/or DPWG clinical guidelines are included.

 The *Gene S* RosaMind PGx Test uses Next-Generation Sequencing (NGS) allowing for simultaneous examination of multiple genes, including those relevant to drug metabolism, response, and potential adverse effects. NGS offers high sensitivity and specificity in detecting genetic variations, including single nucleotide polymorphisms (SNPs), insertions/deletions, copy number variation (CNV) and hybrid variants. Any variant locations without an assigned genotype are reported as "No call”. *1 is the reference allele and is reported by default if the other tested alleles are not detected.

Gene S does not take into account other genetic variants and environmental or social factors that may affect patients' response, including but not limited to environmental factors (e.g., smoking, diet), social and familial factors, various medical conditions, and drug-to-drug interactions.

It's important to note that test results and clinical interpretation may be unreliable for individuals who have undergone or are currently receiving non-autologous blood transfusion, tissue, and/or organ transplant therapy. Additionally, while exceedingly rare, other factors not mentioned above, such as laboratory errors, could potentially impact results.   Note that the exact allele orientation cannot be determined without familial segregation testing.

## Supplementary Material S2. Example Treatment Guide

**[*Insert date here*]**

**[*Insert GP name here*]**

**[*Insert GP practice here*]**

**[*Insert GP practice address here*]**

**RE:** **GENE-YD Study Treatment Guide for [*Insert participant name here*]**

Dear Dr [*Insert GP name here*],

Your patient **[*Insert participant name here*] (DOB:** **[*Insert participate DOB here*])** was recently screened for participation in the Pharmacogenetics in Youth Depression (GENE-YD) Study. The screening process comprised the DSM-5 M.I.N.I. assessment to establish a diagnosis of major depressive disorder. Based on the results of the screening assessments, your patient has been diagnosed with Major Depressive Disorder and has consented to participate in the GENE-YD Study.

The Treatment Guide below has been developed based on the Australian and New Zealand College of Psychiatrists’ Clinical Practice Guidelines for Major Depression (Malhi et al., 2018) and the latest edition of Therapeutic Guidelines: Psychotropic (Version 8). It has been prepared by the GENE-YD Study Treatment Plan Writing Group led by psychiatrist Prof. Sean Hood. Please note that the Treatment Plan is personalised to this patient and is not necessarily applicable to others.

**How to Use This Treatment Guide**

- The list below is a selection of recommended medication(s) for your patient. The list contains recommended first line and second line medications. The second line medications are provided in the case of patients not responding to first line treatment.
- Please select **ONE** drug from the list of first line medications.
- If your patient does not tolerate this medication for any reason, please select another first line treatment listed below.
- If your patient is not responding to (or does not tolerate) the first line treatments, please select a second line treatment listed below.
- Guidance is provided for a suggested starting dose for the first week, and then the standard dose to be taken, typically from week 1 onwards. The medications listed are in alphabetical order, and do not reflect treatment preference.
- **As with all pharmacotherapies, please exercise clinical judgement and monitor your patient’s response as is your usual clinical practice.**
- Please note that the recommended doses may not be standard. The doses may have been adjusted based on your patient’s pharmacogenomic data**.**

| Medication^#^  *List is in alphabetical order, not order of preference.* | Initial Dose (mg) | Ongoing Dose (mg) |
| --- | --- | --- |
| First treatment considered |  |  |
| Fluvoxamine | 50 nocte | 100 nocte |
| Mirtazapine | 15 nocte | 30 nocte |
| Sertraline | 50 mane | 100 mane |
| Second treatment considered |  |  |
| Desvenlafaxine | 50 mane | 100 mane |
| Duloxetine | 30 mane | 60 mane |
| Moclobemide | 150 BD | 300 BD |

*(#): These recommendations are provided under the assumption that any antidepressant your patient is currently taking will be discontinued with appropriate wash-out period as relevant prior to commencing a different antidepressant recommended in this treatment guide, for the treatment of depression.*

**These recommendations do not take into account of potential drug-drug interactions with other concomitant medications your patient may be taking. Usual prescribing precautions apply.**

You should bill your patient as normal: Medicare rebates are claimable as usual, and medications on the PBS will be subsidised by the PBS as normal. Please provide follow-up visits as deemed necessary per your clinical judgment.

If you any questions have any questions about this report, including interpretation and clinical utility, the GENE-YD Study team can be reached at [*Insert study team contact information here*].

Yours Sincerely,

[*Insert signature here]*

[*Insert psychiatrist name, qualification, and position here*]

*NOTE: Treatment Guides were standardized in format and presentation across study arms, with medication options listed alphabetically to minimise systematic differences and reduce the potential for inference of group allocation. Guides were prepared by an unblinded psychiatrist using participants’ demographic, clinical, and pharmacogenetic information, however, no explicit indicators of treatment group were provided to prescribing clinicians. While it is possible that clinicians may have inferred potential PGx-guidance based on the inclusion or exclusion of specific medications, this was not expected to occur consistently, as participants with non-actionable PGx profiles could receive recommendations similar to those in the treatment-as-usual group. Final prescribing decisions were made by the treating clinician based on their clinical judgement.*

### Supplementary Material S3. Bayesian analyses

As an exploratory sensitivity analysis, Bayesian mixed-effects models adjusting for baseline values of the outcome measures were also fitted to account for any baseline group differences.

**Methods**

These models were estimated using weakly informative priors with mild regularization (Normal(0, 0.5) for regression coefficients). Further analyses were conducted using more strongly regularizing priors (Normal(0, 0.3)) to assess the robustness of findings to prior specification, under the assumption that standardized effects would be small to moderate.

**Results**

| Table S1. Bayesian standardised effect size estimates across models with differing prior specifications. Results are presented for models using weakly informative priors (Normal(0, 0.5)) and moderately regularising priors (Normal(0, 0.3)) for regression coefficients. | | | | | | | | | | |
| --- | --- | --- | --- | --- | --- | --- | --- | --- | --- | --- |
| Scale | **Domain** | **Timepoint** | **Posterior median effect size, *g*** | | **95% credible interval (lower)** | | **95% credible interval (upper)** | | **Posterior probability, *P*** | |
|  |  |  | **Weakly informative** | **More regularized** | **Weakly informative** | **More regularized** | **Weakly informative** | **More regularized** | **Weakly informative** | **More regularized** |
| MADRS | Rater-Assessed: Depression | Week 6 | - | - | - | - | - | - | - | - |
|  |  | Week 12 | -0.24 | -0.11 | -0.86 | -0.54 | 0.36 | 0.33 | 0.78 | 0.70 |
| QIDS  SR16 | Self-Report: Depression | Week 6 | -0.13 | -0.06 | -0.75 | -0.48 | 0.52 | 0.36 | 0.65 | 0.61 |
|  |  | Week 12 | -0.41 | -0.20 | -1.06 | -0.60 | 0.23 | 0.22 | 0.89 | 0.83 |
| DASS-21 | Depression | Week 6 | 0.15 | 0.07 | -0.48 | -0.34 | 0.78 | 0.49 | 0.33 | 0.36 |
|  |  | Week 12 | -0.08 | -0.03 | -0.69 | -0.45 | 0.54 | 0.38 | 0.60 | 0.57 |
|  | Anxiety | Week 6 | 0.22 | 0.09 | -0.42 | -0.29 | 0.86 | 0.48 | 0.25 | 0.32 |
|  |  | Week 12 | -0.31 | -0.15 | -0.96 | -0.55 | 0.34 | 0.25 | 0.82 | 0.77 |
|  | Stress | Week 6 | 0.00 | 0.00 | -0.62 | -0.36 | 0.62 | 0.36 | 0.50 | 0.50 |
|  |  | Week 12 | -0.09 | -0.04 | -0.71 | -0.42 | 0.54 | 0.33 | 0.61 | 0.59 |
| AQoL-8D | Total AQoL-8D Score | Week 6 | -0.09 | -0.04 | -0.68 | -0.42 | 0.51 | 0.34 | 0.61 | 0.58 |
|  |  | Week 12 | -0.23 | -0.12 | -0.82 | -0.52 | 0.36 | 0.27 | 0.79 | 0.73 |
| ASEC | Total ASEC Score for Antidepressant-related Side Effects | Week 6 | 0.03 | -0.00 | -0.63 | -0.41 | 0.67 | 0.40 | 0.46 | 0.51 |
|  |  | Week 12 | -0.33 | -0.17 | -0.98 | -0.57 | 0.32 | 0.22 | 0.84 | 0.80 |
| Note: Standardised effect sizes are reported as Hedges’ *g* from Bayesian mixed-effects models. Negative values indicate greater improvement in the PGx-Guided group relative to treatment-as-usual. Credible intervals represent 95% posterior credible intervals. Posterior probabilities indicate the probability that the PGx-guided group showed greater improvement. | | | | | | | | | | |

## Supplementary Material S4. Baseline assessment scores

| Table S2. Baseline scores for rater-assessed and self-report measures in the GENE-YD Study. | | | |
| --- | --- | --- | --- |
| Scale | **Total** | **Intervention Groups** | |
|  | **All (*N* = 39)** | **TAU (*n* = 20)** | **PGx-Guided (*n* =19)** |
| MADRS total score (0-60), mean (SD) | 24.1 (10.4) | 22.6 (11.4) | 25.8 (9.2) |
| QIDS-SR16 total score (0-27), mean (SD) | 12.8 (4.3) | 12.8 (4.9) | 12.8 (3.7) |
| DASS-21 depression score (0-21), mean (SD) | 8.1 (4.0) | 7.9 (4.1) | 8.3 (3.9) |
| DASS-21 anxiety score (0-21), mean (SD) | 6.8 (4.6) | 7.3 (4.9) | 6.4 (4.3) |
| DASS-21 stress score (0-21), mean (SD) | 9.3 (3.8) | 9.3 (4.3) | 9.3 (3.2) |
| Perceived quality of life (AQoL-8D total; 35-175), mean (SD) | 91.3 (16.0) | 90.7 (16.5) | 91.9 (15.9) |
| Independent living (4-20) | 8.3 (2.7) | 8.5 (2.8) | 8.2 (2.5) |
| Senses (3-15) | 5.6 (1.6) | 5.4 (1.6) | 5.8 (1.6) |
| Pain (3-15) | 5.9 (2.6) | 6.2 (2.7) | 5.6 (2.6) |
| Mental health (8-40) | 23.7 (4.2) | 23.7 (4.1) | 23.6 (4.3) |
| Happiness (4-20) | 12.3 (2.3) | 12.0 (2.4) | 12.6 (2.2) |
| Self-Worth (3-15) | 9.1 (2.3) | 8.8 (2.4) | 9.3 (2.4) |
| Coping (3-15) | 9.2 (2.0) | 9.0 (2.2) | 9.5 (1.8) |
| Relationships (7-35) | 17.2 (4.3) | 17.2 (4.3) | 17.3 (4.4) |
| Number of side effect presentations, mean (SD) | 3.2 (3.0) | 3.8 (3.2) | 2.4 (2.8) |
| Side effect burden (0-63), mean (SD) | 4.6 (4.7) | 5.5 (4.7) | 3.8 (4.6) |
| Number of side effects with severity level 1, *n* (%) | 29 (74.4) | 16 (80.0) | 13 (68.4) |
| Number of side effects with severity level 2, *n* (%) | 20 (51.3) | 12 (60.0) | 8 (42.1) |
| Number of side effects with severity level 3, *n* (%) | 6 (15.4) | 3 (15.0) | 3 (15.8) |
| MARS-5 score (5-25), mean (SD) | 22.2 (2.9) | 22.6 (2.8) | 21.8 (3.0) |
| Hours spent in social activities in the last 7 days, mean (SD) | 14.2 (13.6) | 18.4 (17.7) | 9.7 (4.9) |
| Hours spent studying in the last 7 days, mean (SD) | 12.5 (17.7) | 17.1 (21.0) | 7.6 (12.1) |
| Hours spent in unpaid work in the last 7 days, mean (SD) | 0.6 (1.9) | 0.6 (1.9) | 0.6 (1.9) |
| Hours spent in a caring role in the last 7 days, mean (SD) | 4.6 (23.5) | 0.7 (2.3) | 8.7 (33.6) |
| Hours spent in paid work in the last 7 days, mean (SD) | 10.9 (11.5) | 6.8 (9.9) | 15.2 (11.8) |
| Times visited the GP in the last 12 months, mean (SD) | 7.5 (8.1) | 7.4 (5.9) | 7.6 (10.0) |
| Currently employed, (yes), *n* (%) | 23 (59.0) | 9 (45.0) | 14 (73.7) |
| Work absenteeism (%), mean (SD) | 3.2 (8.1) | 0.4 (1.1) | 5.0 (10.0) |
| Work presenteeism (%), mean (SD) | 33.9 (22.5) | 38.9 (22.6) | 30.7 (22.7) |
| Overall work productivity loss (%), mean (SD) | 35.8 (23.4) | 39.1 (22.6) | 33.7 (24.5) |
| Activity impairment (%), mean (SD) | 41.4 (25.1) | 42.6 (24.5) | 40.0 (26.3) |

## Supplementary Material S5. Prescription congruency

Pharmacogenomic (PGx) prescribing recommendations, as reported by the *Gene S* ‘RosaMind’ PGx Solution, classify antidepressants into categories reflecting the expected impact of genetic variation on medication exposure or response (Roberts et al., 2025). These categories, *no genetic concerns*, *consider increasing/decreasing dose*, and *advised to avoid*, indicate whether standard dosing is appropriate or whether dose modification is recommended based on an individual’s PGx phenotype. Standard dosing guidelines for each antidepressant were defined according to Table 1 in Roberts et al. (2025). In the GENE-YD study, congruency aimed to assess whether participants’ prescribed medications and doses aligned with the recommendations provided in their personalised PGx report.

**Methods**

Congruency was assessed with medications taken by participants prior to the study prescription, at baseline, and at Week 12 to capture any congruency changes with medication and dosage adjustments during the follow-up period. Congruency was determined by comparing each participant’s prescribed antidepressant and dose against their PGx phenotype as identified in their individual PGx report. For each medication:

- Congruent prescribing was defined as:
  1. Medications classified as “*no genetic concerns*” prescribed within standard dosing guidelines; or
  2. Medications classified as “*consider increasing dose*” or “*consider decreasing dose*” with doses appropriately adjusted from the standard guidelines.
  3. A prescription with fluoxetine or mirtazapine, given the lack of current ClinPGx guidelines to suggest any gene-drug interactions.
- Incongruent prescribing was defined as:
  1. Medications classified as “*no genetic concerns*” prescribed with dose modifications to the standard dosing guidelines; or
  2. Medications classified as “*consider increasing dose*” or “*consider decreasing dose*” prescribed without the recommended PGx-based dose adjustment; or
  3. Medications classified as “*advised to avoid*” prescribed.

A binary congruent (1) / incongruent (0) variable was created for analysis and grouped frequencies were compared between study arms (PGx-Guided vs treatment-as-usual [TAU]).

## Supplementary Material S6. Gatekeeper analysis

In PGx studies, not all participants carry variants that would necessitate changes to standard prescribing. The gatekeeper analysis focuses on participants with actionable PGx variants, excluding those with only normal metaboliser phenotypes for cytochrome P450 genes CYP2C19 and CYP2D6 (Swen et al., 2023). This approach isolates the subset of participants for whom PGx-guided recommendations could meaningfully influence prescribing decisions. By limiting analyses to this group, the gatekeeper analysis tests whether observed effects are driven by participants with clinically relevant PGx profiles rather than the overall sample.

**Methods**

Participants were filtered to exclude individuals with normal PGx phenotypes for CYP2C19 and CYP2D6 and no minor or major prescribing considerations (*n* = 3). All outcome analyses, including symptom improvement, response, and remission rates, and quality of life assessments, were then rerun in this reduced sample (*N* = 36). Statistical procedures mirrored the primary analyses but were restricted to participants with actionable PGx findings to determine whether the presence of these variants influenced key study outcomes.

**Results**

Table S3 provides a full comprehensive breakdown of the outcomes from the gatekeeper analysis.

| Table S3. All measures over time for each scale and domain used within the GENE-YD Study’s gatekeeper analysis. Timepoint measures presented as group means (SEM) and change (Δ) to baseline and between group as mean difference (95% CI). | | | | | | | | |
| --- | --- | --- | --- | --- | --- | --- | --- | --- |
|  |  |  | **Timepoint** | | | | | |
| **Scale** | **Domain** |  | **Baseline** | **Week 6** | **Δ6** | **Week 12** | **Δ12** | **Between Group Δ12_PGx-TAU_** |
| MADRS | Rater-Assessed: Depression | TAU (*n* = 18) | 22.3 (2.4) | - | - | 14.8 (2.4) | -7.5 (-14.2, -0.8) | - |
|  |  | PGx-Guided (*n* = 18) | 25.4 (2.4) | - | - | 13.4 (2.4) | -12 (-18.6, -5.4) | -4.5 (-13.9, 4.9) |
| QIDS-SR16 | Self-Report: Depression | TAU (*n* = 18) | 12.7 (1.0) | 10.0 (1.1) | -2.7 (-5.5, 0.2) | 9.5 (1.1) | -3.2 (-6.1, -0.3) | - |
|  |  | PGx-Guided (*n* = 18) | 12.6 (1.0) | 9.6 (1.0) | -3.0 (-5.8, -0.2) | 8.1 (1.0) | -4.5 (-7.3, -1.7) | -1.3 (-5.3, 2.7) |
| DASS-21 | Depression | TAU (*n* = 18) | 8.0 (0.9) | 6.4 (1.0) | -1.6 (-4.3, 1.0) | 6.6 (1.0) | -1.4 (-4.0, 1.3) | - |
|  |  | PGx-Guided (*n* = 18) | 7.7 (0.90 | 7.4 (0.9) | -0.3 (-2.9, 2.3) | 6.2 (0.9) | -1.5 (-4.1, 1.1) | -0.1 (-3.8, 3.5) |
|  | Anxiety | TAU (*n* = 18) | 7.7 (0.8) | 5.6 (0.8) | -2.1 (-4.4, 0.2) | 5.1 (0.8) | -2.6 (-4.9, -0.3) | - |
|  |  | PGx-Guided (*n* = 18) | 6.2 (0.8) | 5.8 (0.8) | -0.4 (-2.7, 1.8) | 3.8 (0.8) | -2.4 (-4.7, -0.2) | 0.2 (-3.1, 3.4) |
|  | Stress | TAU (*n* = 18) | 9.2 (0.9) | 8.2 (0.9) | -1.0 (-3.5, 1.5) | 7.5 (0.9) | -1.7 (-4.2, 0.8) | - |
|  |  | PGx-Guided (*n* = 18) | 9.4 (0.9) | 8.5 (0.9) | -0.9 (-3.4, 1.6) | 7.1 (0.9) | -2.3 (-4.7, 0.2) | -0.6 (-4.1, 2.9) |
| AQoL-8D | Independent Living | TAU (*n* = 18) | 8.5 (0.7) | 8.2 (0.7) | -0.3 (-2.1, 1.6) | 7.8 (0.7) | -0.7 (-2.5, 2.1) | - |
|  |  | PGx-Guided (*n* = 18) | 8.1 (0.7) | 7.7 (0.7) | -0.4 (-2.3, 1.4) | 7.5 (0.7) | -0.6 (-2.4, 1.2) | 0.1 (-2.5, 2.6) |
|  | Senses | TAU (*n* = 18) | 5.6 (0.4) | 4.9 (0.4) | -0.6 (-1.8, 0.6) | 5.8 (0.4) | 0.2 (-1.0, 1.4) | - |
|  |  | PGx-Guided (*n* = 18) | 5.7 (0.4) | 5.8 (0.4) | -0.1 (-1.2, 1.1) | 5.7 (0.4) | 0.0 (-1.2, 1.2) | -0.2 (-1.9, 1.4) |
|  | Pain | TAU (*n* = 18) | 5.8 (0.5) | 5.2 (0.5) | -0.6 (-2.0, 0.8) | 5.6 (0.5) | -0.2 (-1.6, 1.2) | - |
|  |  | PGx-Guided (*n* = 18) | 5.8 (0.5) | 4.9 (0.5) | -0.9 (-2.3, 0.5) | 5.1 (0.5) | -0.7 (-2.1, 0.7) | -0.5 (-2.2, 1.7) |
|  | Mental Health | TAU (*n* = 18) | 23.4 (1.0) | 21.7 (1.0) | -1.7 (-4.5, 1.0) | 20.4 (1.0) | -3.0 (-5.7, -0.2) | - |
|  |  | PGx-Guided (*n* = 18) | 23.4 (1.0) | 21.7 (1.0) | -1.7 (-4.4, 1.1) | 20.4 (1.0) | -3.0 (-5.7, -0.3) | 0.0 (3.9, 3.9) |
|  | Happiness | TAU (*n* = 18) | 12.0 (0.6) | 11.2 (0.6) | -0.8 (-2.4, 0.8) | 11.2 (0.6) | -0.8 (-2.4, 0.8) | - |
|  |  | PGx-Guided (*n* = 18) | 12.4 (0.6) | 11.6 (0.6) | -0.8 (-2.4, 0.8) | 11.0 (0.6) | -1.4 (-3.0, 0.2) | -0.6 (-2.9, 1.6) |
|  | Self-Worth | TAU (*n* = 18) | 8.7 (0.4) | 7.6 (0.4) | -1.1 (-2.3, 0.2) | 7.8 (0.4) | -0.9 (-2.1, 0.3) | - |
|  |  | PGx-Guided (*n* = 18) | 9.0 (0.4) | 8.3 (0.4) | -0.7 (-1.9, 0.5) | 7.5 (0.4) | -1.5 (-2.7, -0.3) | -0.6 (-2.3, 1.1) |
|  | Coping | TAU (*n* = 18) | 9.1 (0.5) | 8.4 (0.5) | -0.7 (-2.0, 0.6) | 8.1 (0.5) | -1.0 (-2.3, 0.3) |  |
|  |  | PGx-Guided (*n* = 18) | 9.4 (0.5) | 9.1 (0.5) | -0.3 (-1.6, 1.0) | 8.2 (0.5) | -1.2 (-2.5, 0.1) | -0.2 (-2.1, 1.6) |
|  | Relationships | TAU (*n* = 18) | 17.4 (1.0) | 16.1 (1.0) | -1.3 (-4.1, 1.4) | 15.5 (1.0) | -1.9 (-4.7, 0.8) | - |
|  |  | PGx-Guided (*n* = 18) | 16.9 (1.0) | 15.5 (1.0) | -1.4 (-4.1, 1.3) | 15.0 (1.0) | -1.9 (-4.6. 0.8) | 0.0 (-3.9, 3.9) |
|  | Total AQoL-8D Score | TAU (*n* = 18) | 90.5 (3.7) | 83.4 (3.8) | -7.1 (-17.6, 3.4) | 82.3 (3.8) | -8.2 (-18.7, 2.3) | - |
|  |  | PGx-Guided (*n* = 18) | 90.7 (3.7) | 84.6 (3.7) | -6.1 (-16.5, 4.3) | 80.3 (3.7) | -10.3 (-20.7, 0.0) | -2.1 (-16.9, 12.6) |
| ASEC | Frequency | TAU (*n* = 18) | 3.8 (0.9) | 5.3 (0.9) | 1.5 (-0.9, 3.9) | 6.2 (0.9) | 2.4 (0.0, 4.8) |  |
|  |  | PGx-Guided (*n* = 18) | 2.4 (0.9) | 6.8 (0.9) | 4.4 (2.0, 6.7) | 4.8 (0.9) | 2.4 (0.0, 4.8) | 0.0 (-3.4, 3.4) |
|  | Severity | TAU (*n* = 18) | 5.5 (1.0) | 4.1 (1.0) | -1.4 (-4.1, 1.2) | 4.2 (1.0) | -1.3 (-3.9, 1.4) | - |
|  |  | PGx-Guided (*n* = 18) | 3.8 (1.0) | 3.7 (1.0) | -0.1 (-2.8, 2.4) | 2.7 (1.0) | -1.1 (-3.8, 1.4) | 0.1 (-3.6, 3.8) |
| MARS-5 | Medication Adherence | TAU (*n* = 18) | 22.3 (0.6) | 23.0 (0.6) | 0.7 (-1.0, 2.3) | 22.5 (0.6) | 0.2 (-1.5, 1.8) | - |
|  |  | PGx-Guided (*n* = 18) | 21.7 (0.6) | 22.7 (0.6) | 1.0 (-0.6, 2.6) | 21.8 (0.6) | 0.1 (-2.5, 0.7) | -0.3 (-2.3, 2.3) |
| LCQ | Social Participation | TAU (*n* = 18) | 20.0 (3.1) | - | - | 22.3 (3.2) | 2.3 (-6.5, 11.0) | - |
|  |  | PGx-Guided (*n* = 18) | 9.7 (3.1) | - | - | 15.4 (3.1) | 5.7 (-3.0, 14.3) | 3.4 (-8.9, 15.7) |
|  | Study Participation | TAU (*n* = 18) | 17.8 (3.6) | - | - | 8.5 (3.7) | -9.3 (-19.6, 0.9) | - |
|  |  | PGx-Guided (*n* = 18) | 7.8 (3.6) | - | - | 11.5 (3.6) | 3.7 (-6.4, 13.8) | 13.1 (-1.3, 27.5) |
|  | Unpaid Work Participation | TAU (*n* = 18) | 0.4 (0.6) | - | - | 1.7 (0.6) | 1.3 (-0.5, 3.0) | - |
|  |  | PGx-Guided (*n* = 18) | 0.7 (0.6) | - | - | 0.2 (0.6) | -0.5 (-2.2, 1.2) | -1.8 (-4.2, 0.7) |
|  | Carer Participation | TAU (*n* = 18) | 0.8 (4.7) | - | - | 4.7 (6.0) | 3.9 (-10.9, 18.7) | - |
|  |  | PGx-Guided (*n* = 18) | 9.2 (4.7) | - | - | 1.6 (7.5) | -7.6 (-24.8, 9.6) | -11.5 (-34.2, 11.2) |
|  | Paid Work Participation | TAU (*n* = 18) | 7.5 (2.5) | - | - | 14.2 (3.2) | 6.7 (-1.3, 14.8) | - |
|  |  | PGx-Guided (*n* = 18) | 16.1 (2.5) | - | - | 22.0 (2.9) | 5.9 (-1.5, 13.4) | -0.8 (-11.7, 10.2) |
|  | GP Visits:  Annually | TAU (*n* = 18) | 7.2 (1.4) | - | - | 7.4 (1.5) | 0.2 (-3.9, 4.2) | - |
|  |  | PGx-Guided (*n* = 18) | 7.2 (1.4) | - | - | 6.0 (1.4) | -1.2 (-5.2, 2.8) | -1.4 (-7.1, 4.3) |
| WPAI | Absenteeism | TAU (*n* = 18) | 0.4 (2.8) | - | - | 5.5 (2.6) | 5.1 (-2.3, 12.6) | - |
|  |  | PGx-Guided (*n* = 18) | 5.0 (2.2) | - | - | 3.0 (2.1) | -2.0 (-8.0, 4.0) | -7.2 (-16.7, 2.4) |
|  | Presenteeism | TAU (*n* = 18) | 38.9 (6.9) | - | - | 35.0 (6.6) | -3.9 (-22.6, 14.8) | - |
|  |  | PGx-Guided (*n* = 18) | 30.7 (5.5) | - | - | 18.0 (5.4) | -12.7 (-27.8, 2.4) | -8.8 (-32. 9, 15.2) |
|  | Work Impairment | TAU (*n* = 18) | 39.1 (7.5) | - | - | 33.6 (6.8) | -5.5 (-25.4, 14.4) | - |
|  |  | PGx-Guided (*n* = 18) | 33.7 (6.0) | - | - | 20.3 (5.8) | -13.4 (-29.9, 3.0) | -8.0 (-33.7, 17.8) |
|  | Activity Impairment | TAU (*n* = 18) | 44.7 (6.2) | - | - | 35.6 (6.3) | -9.1 (-26.4, 8.2) | - |
|  |  | PGx-Guided (*n* = 18) | 40.0 (6.0) | - | - | 27.2 (6.0) | -12.8 (-29.3, 3.8) | -3.7 (-27.7, 20.3) |

## Supplementary Reference List

Roberts, B., Cooper, Z., Miljevic, A., Stanley, S. H., Majda, B. T., Collins, K. R. L., Baltic, S., Lindsey-Temple, S. E., Akkari, P. A., Hood, S. D., & Rodger, J. (2025). PharmacoGENEtics in Youth Depression (GENE-YD) Study protocol: a pilot triple-blinded randomised controlled trial using pharmacogenetics to tailor antidepressant treatment in young Australians. *BMJ Open*, *15*(11), e103644. <https://doi.org/10.1136/bmjopen-2025-103644>

Swen, J. J., van der Wouden, C. H., Manson, L. E., Abdullah-Koolmees, H., Blagec, K., Blagus, T., Böhringer, S., Cambon-Thomsen, A., Cecchin, E., Cheung, K. C., Deneer, V. H., Dupui, M., Ingelman-Sundberg, M., Jonsson, S., Joefield-Roka, C., Just, K. S., Karlsson, M. O., Konta, L., Koopmann, R.,…Guchelaar, H. J. (2023). A 12-gene pharmacogenetic panel to prevent adverse drug reactions: an open-label, multicentre, controlled, cluster-randomised crossover implementation study. *Lancet*, *401*(10374), 347–356. <https://doi.org/10.1016/s0140-6736(22)01841-4>
